# Hallux Limitus Influence on Plantar Pressure Variations during the Gait Cycle: A Case-Control Study

**DOI:** 10.3390/bioengineering10070772

**Published:** 2023-06-27

**Authors:** Claudia Cuevas-Martínez, Ricardo Becerro-de-Bengoa-Vallejo, Marta Elena Losa-Iglesias, Israel Casado-Hernández, Emmanuel Navarro-Flores, Laura Pérez-Palma, João Martiniano, Juan Gómez-Salgado, Daniel López-López

**Affiliations:** 1 Research, Health, and Podiatry Group, Department of Health Sciences, Faculty of Nursing and Podiatry, Industrial Campus of Ferrol, Universidade da Coruña, 15403 Ferrol, Spain; claudia.cuevas@udc.es (C.C.-M.); daniellopez@udc.es (D.L.-L.); 2 Departament de Podologia, Facultat de Medicina i Ciències de la Salut, Universitat de Barcelona, 08036 Barcelona, Spain; lperez@ub.edu; 3 Facultad de Enfermería, Fisioterapia y Podología, Universidad Complutense de Madrid, 28040 Madrid, Spain; ribebeva@ucm.es; 4 Faculty of Health Sciences, Universidad Rey Juan Carlos, 28922 Alcorcon, Spain; marta.losa@urjc.es; 5 Frailty Research Organizaded Group (FROG), Department of Nursing, Faculty of Nursing and Podiatry, University of Valencia, 46010 Valencia, Spain; emmanuel.navarro@uv.es; 6 Escola Superior de Saúde da Cruz Vermelha Portuguesa, 1300-125 Lisbon, Portugal; jmartiniano@esscvp.eu; 7 Department of Sociology, Social Work and Public Health, Faculty of Labour Sciences, University of Huelva, 21004 Huelva, Spain; salgado@uhu.es; 8 Health and Safety Postgraduate Programme, Universidad Espíritu Santo, Guayaquil 092301, Ecuador

**Keywords:** gait analysis, hallux limitus, school age, plantar pressure

## Abstract

Background: Hallux limitus is a common foot disorder whose incidence has increased in the school-age population. Hallux limitus is characterized by musculoskeletal alteration that involves the metatarsophalangeal joint causing structural disorders in different anatomical areas of the locomotor system, affecting gait patterns. The aim of this study was to analyze dynamic plantar pressures in a school-aged population both with functional hallux and without. Methods: A full sample of 100 subjects (50 male and 50 female) 7 to 12 years old was included. The subjects were identified in two groups: the case group (50 subjects characterized as having hallux limitus, 22 male and 28 female) and control group (50 subjects characterized as not having hallux limitus, 28 male and 22 female). Measurements were obtained while subjects walked barefoot in a relaxed manner along a baropodometric platform. The hallux limitus test was realized in a seated position to sort subjects out into an established study group. The variables checked in the research were the surface area supported by each lower limb, the maximum peak pressure of each lower limb, the maximum mean pressure of each lower limb, the body weight on the hallux of each foot, the body weight on the first metatarsal head of each foot, the body weight at the second metatarsal head of each foot, the body weight at the third and fourth metatarsal head of each foot, the body weight at the head of the fifth metatarsal of each foot, the body weight at the midfoot of each foot, and the body weight at the heel of each foot. Results: Non-significant results were obtained in the variable of pressure peaks between both study groups; the highest pressures were found in the hallux with a *p*-value of 0.127 and in the first metatarsal head with a *p*-value 0.354 in subjects with hallux limitus. A non-significant result with a *p*-value of 0.156 was obtained at the second metatarsal head in healthy subjects. However, significant results were observed for third and fourth metatarsal head pressure in healthy subjects with a *p*-value of 0.031 and regarding rearfoot pressure in subjects with functional hallux limitus with a *p*-value of 0.023. Conclusions: School-age subjects with hallux limitus during gait exhibit more average peak plantar pressure in the heel and less peak average plantar pressure in the third and fourth metatarsal head as compared to healthy children aged between 7 and 12 years old.

## 1. Introduction

Hallux limitus (HL) is a limitation of the dorsiflexion movement (DF) of the first metatarsophalangeal joint (IMTFJ) [1,2]. Functional hallux limitus (HL) was determined as the restriction of the IMTFJ motion in weight bearing and a normal IMTFJ motion in non-weight bearing [3]; HL produces limitations in gait patterns because of a restriction of closed-kinetic-chain joint motion causing functional limitations during the final phase of gait due to the blockage of the third rocker [4,5,6]. The blockage of the third rocker generates a variation of the axis of the first radius (1R) in the sagittal plane, causing a compensatory mechanism that helps to advance the center of mass along the extrinsic and intrinsic structures of the foot in order to improve the gait and finish it with the propulsion phase. Consequently, in order to improve the joint movement in the last phase of gait, secondary compensations generate a greater or lesser plantar pressure which can be analyzed with a baropodometric platform [6].

Pathologies such as HL, hallux valgus or hallux rigidus are secondary to a wrong position of the axis of the first radius in the sagittal plane [5]. This disorder can be balanced either distally in the interphalangeal joint or proximally in the midtarsal joints or even in the subtalar joint [7,8]. In addition, the IMTFJ disfunction motion in the sagittal plane produced by other biomechanical disorders does not cause local symptoms in the affected joint, as metatarsalgia, tendonitis or sesamoiditis, but can also produce symptoms in the retro malleolar aspect of the flexor hallucis longus tendon, knee and lumbar region [9,10].

A proper DF movement of the IMTJ in motion is necessary to stabilize the foot during the final contact phase of gait and to be effective [1]. If gait stabilization is not achieved and the different gait phases are inefficient, it will generate, in addition to a secondary pathology, a greater energy and functional expenditure during the gait, generating disorders on the muscle-ligamentous integrity of the body structures [11,12,13].

The adult population suffering from HL exhibit compensations that have been observed secondary to the limitation of IMT such as trunk forwarding to advance the center of gravity walking, beside decreasing the ground reactive forces (GRFs) that limit IMTJ motion and reduce the propulsion of the foot. In addition, a decrease extension movement of the hip that supports the lower limb during the gait pattern generates a lack of contraction of the biceps femoris muscle, producing a blocking of the sacroiliac joint and hyperlordosis that is caused by a prolonged muscle activation of the psoas iliacus and quadratus lumborum muscles due to the inability to counteract the action of the hip extensor muscles, causing finally an early heel elevation. Moreover, during the initial contact of the leg in support in the gait phase, the leg will be in flexion to balance the lack of hip extension [14,15].

Dynamic plantar pressure measurements between HL, hallux valgus and non-pathologic feet in the adult population have been previously studied and significant results have been obtained between the two groups, showing a higher plantar pressure under hallux, lesser toes and third and fourth metatarsal heads in subjects with HL [16].

As any restriction of motion should be compensated by other anatomical points, either proximal or distal to the initial block, it is essential to know the initial pathology of the same in IMTFJ, known as hallux limitus at school age, in order to prevent later compensations in adulthood.

The purpose of this study was to analyze the dynamic plantar pressure between subjects who have hallux limitus and subjects without hallux limitus in school age. The research hypothesis was that children in school age who have hallux limitus generate higher plantar pressures under the hallux than subjects without hallux limitus.

## 2. Material and Methods

### 2.1. Study Design

A case-control study was performed from January 2022 to February 2023 and it was carried out to analyze the plantar pressure variations in a school-age population with and without HL in a dynamic gait. This research followed all the criteria of the Strengthening the Reporting of Observational Studies in Epidemiology (STROBE) guidelines [17].

The research was conducted by the University of Barcelona Ethics Committee (consent no. IRB00003099). All the procedures were implemented according to all current regulations on human experimentation, as well as the Declaration of Helsinki and Organic Law 3/2018 of 5 December on protection of personal data and guarantee of digital rights [18].

An expert podiatrist in biomechanics with 10 years’ experience screened all the subjects in advance. The subjects’ were recruited in different private clinics by the same podiatrist.

The inclusion criteria were the following: (1) subjects older than 6 years and younger than 12 years; (2) healthy subjects without musculoskeletal disturbances or foot soreness; (3) subjects with no lower limb surgical intervention; (4) subjects with and without HL; (5) parents agreed to participation in the study and signed informed consent.

The exclusion criteria were the following: (1) subjects under 6 or over 12 years of age; (2) subjects who had suffered any pain or significant foot disorders; (3) subjects under medical treatment could affect the data acquisition; (4) subjects with musculoskeletal injuries or neurological disorders; (5) subjects with hypermobility syndrome; (6) subjects with an IMTFJ angle value lower than 10^0^ flexing the ankle; (7) subjects who refused to adhere to the guidelines to participation in the study.

The levels of confidence, the potential groups of same size and the estimation of the sample size were analyzed using Epidat version 4.2 software (Consellería de Sanidade, Xunta de Galicia, Spain; Pan American Health Organization (PAHO-WHO); University CES, Colombia). In addition, an 80% statistical power analysis with a β error = 20%, an α error = 0.05 and a two tailed test were required to ensure statistical confidence. Finally, a full sample size of 100 children aged between 7 and 12 years old was composed. The groups were classified as follows: 50 subjects with HL and 50 healthy subjects.

### 2.2. Sociodemographic Data

A total sample of 100 subjects with an age range between seven and twelve years old and a mean age ± SD of 9.62 ± 1.37 years were included. From the 100 subjects recruited, 50 exhibited HL and the remainder did not have diseases and were the control group. The full sample size was composed of 50 male and 50 female subjects. Quantitative sociodemographic, anthropometric and descriptive outcomes are shown in Table 1 including age, weight, height, body mass index (BMI), sex and foot size.

Table 1 does not show statistically significant differences among groups regarding sociodemographic and quantitative descriptive data.

### 2.3. Procedure

Once subjects agreed to take part in the research, the podiatrist checked them to make sure the inclusion criteria were achieved. Subsequently, the subjects were barefoot to be measured and weighed. Next, the podiatrist checked the hallux joint mobility. To assess this, the clinician maintained the subtalar joint neutrality and the maximum ankle DF was performed with knee extended [19] and to verify that the DF of the IMTFJ was greater than 10^0^, a goniometer was used [4].

The hallux limitus test was conducted with the subject in a resting position, and the clinician had to apply strength under the I metatarsal head (IMTH) with the non-resistive hand, and with the active hand had to perform a DF movement of the hallux. The force applied under the IMTH was approximately the same as used to carry out the DF of the hallux, which was followed by an IMTFJ dorsiflexion, IMTH plantarflexion, forefoot pronation, rearfoot supination, and windlass mechanism outset. Furthermore, when many movements were necessary to realize an effective propulsion, it was considered a negative test (HL−). On the other hand, when subjects had HL in which the forces applied were not balanced and the force needed to realize IMTFL dorsiflexion applied under IMTH was greater than usual [3], it was considered a positive test (HL+).

To conduct the research, a baropodometric portable platform composed of 1600 resistive sensors was used. Regarding the technical specification, the size of each sensor was 10 mm × 10 mm, the sample rate was from 100 Hz to 150 Hz via Wi-Fi and the interface used to connect with the laptop was a USB^®^; a dual amplifier was used to acquire plantar pressure data in dynamics. The portable pressure platform dimension was 565 mm length × 612 mm width × 22 mm height with a detection area of 400 × 400 mm composed of autocalibrated resistive sensors with 8 mm thickness. Autocalibration was conducted before each onset.

To transfer data from the portable pressure platform to a laptop, a USB was used. The software used to interpret the data from the platform manufacturer was T-Plate^®^ (Herbitas, Foios, Valencia, Spain).

Dynamic data plantar pressure was obtained by the same clinician. In the dynamic test, subjects walked barefoot on the platform in a relaxed condition.

If the subject performed some altered movement during the data acquisition, the final data were erased, and the trial was once again repeated. If the subject was uncomfortable or restless, data were discarded. Three dynamic data acquisitions were taken for each subject.

**Figure 1:**
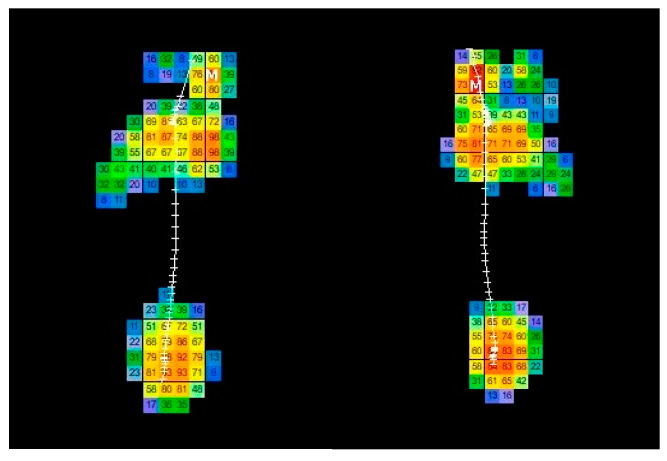
Descriptive analysis of the plantar pressure mapping in dynamic phase with T-Plate software.

This process was repeated until the subject reproduced a relaxed and comfortable gait. Four trials were recorded for each foot and the average calculated via the software was used for the analysis (Figure 1).

The T-Plate^®^ software was used to collect and manage the surface area supported by each limb (cm^2^), the maximum peak pressure of each limb (kPa), the average peak pressure of each limb (kPa), the body weight in the hallux of each foot (%), the body weight in the first metatarsal head of each foot (%), the body weight in the second metatarsal head of each foot (%), the body weight in the third and fourth metatarsal head of each foot (%), the body weight in the fifth metatarsal head of each foot (%), the body weight in the midfoot of each foot and the body weight in the heel of each foot (%).

**Figure 2:**
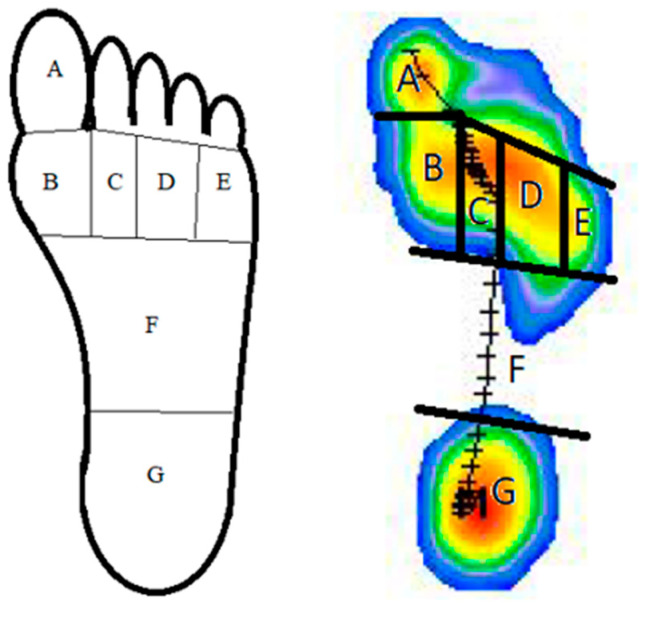
Foot division into seven regions to determine peak pressure location with T-Plate software. Region A: hallux; region B: first metatarsal head; region C: second metatarsal head; region D: third and fourth metatarsal head; region E: fifth metatarsal head; region F: midfoot; region G: heel.

The clinician divided the foot into seven regions (Figure 2) and compared the maximum peak pressures of each region between the two groups. To obtain the location of the pressure peaks of each foot, the body weight in the hallux, the body weight in the first metatarsal head, the body weight in the second metatarsal head, the body weight in the third and fourth metatarsal head, the body weight in the fifth metatarsal head, the body weight in the midfoot and the body weight in the heel were measured.

The maximum plantar pressure was analyzed in seven foot regions while the subject walked on the platform as follows: In region A, the hallux; in region B, the first metatarsal head; in region C, the second metatarsal head; in region D, the third and fourth metatarsal head; in region E, the fifth metatarsal head; in region F, the midfoot; in the region G, the heel (Figure 2).

### 2.4. Statistical Analysis

Parametric outcomes were as follows: (1) mean; (2) ± standard deviation (SD); (3) range values (maximum and minimum). Normality outcomes were checked according to the Kolmogorov–Smirnov test for all the variables on static plantar measuring (*p* > 0.05). Independent t-tests were performed for the variables with normal distribution. The Mann–Whitney “U” test for non-parametric phenomena was used to contrast groups with or without HL. In addition, a *p* value < 0.05 with a 95% confidence interval was considered statistically significant.

To perform all the statistical analyses, the software used was SPSS 19.0.

## 3. Results

### Measured Data’s Principal Outcomes

According to the findings in Table 2, the variable body weight of the third and fourth metatarsal heads (left/right) with *p* = 0.031 and the variable body weight on the heel (left/right) with *p* = 0.023 showed a statistically significant difference between the HL and the control group. As for the other variables, none of them showed statistically significant differences.

## 4. Discussion

This study is the first that has been conducted with school-aged subjects with HL disorders, and plantar pressure variations in dynamic conditions were measured. The main goal of this research was to analyze the plantar pressure in dynamic conditions in a population of children with hallux limitus disorder with aged between seven and twelve years old.

The authors performed similar research and concluded that the gait cycle creates a complex and unconscious motor pattern that allows people to develop, interact and participate in daily activities [20,21,22]. Gibson et al. [22] also specify that gait development has a temporal acquisition process and Samson et al. also indicate that biomechanical growing changes of joint dynamics occur at the age of 4 years old in the ankle joint, and in the knee and hip joint among 6 and 7 years old, respectively; Ito T et al. [20] and Samson et al. [23] determined that in healthy individuals, the biomechanical joint parameters start to stabilize between 5 and 7 years of age. For this reason, the subjects who participated in the study were aged between 7 and 12 years old, so that gait was already integrated as an unconscious activity, reducing the bias due to dynamic immaturity.

Bryant et al. [16] compared plantar pressures in adults with hallux valgus, hallux limitus and in normal IMTJ motion feet, and the results showed rise peak plantar pressures under the first, second and third MTH in non-pathologic feet subjects and under hallux and third, fourth and fifth MTH in hallux limitus feet subjects. However, in our research, we found non-significant results in the location of plantar pressure peaks between both study groups, where there were higher pressure points at hallux and IMTH in subjects with HL. There were significant results showing increased pressure at third and fourth MTH in healthy subjects and higher pressure in the rearfoot in subjects with HL. Thus, data showed non-statistically significant differences between groups; subjects with HL showed a rise in pressure peaks under the hallux and IMTH compared to the control group.

Findlow et al. [24] reported the characteristics of the kinematic differences between flexible feet and feet with greater restriction of movement, the former being feet with heel eversion and a low medial arch. The kinematic difference appears mainly in the forefoot; in flexible feet, the GRFs advance medially and due to forefoot eversion a DF movement of the IMTH is generated; the ground force reaction passes to the second MTH, generating a greater pressure on this and also on the third MTH. On the other hand, supinated feet lateralize the GRF. DF movement of the fifth metatarsal was limited and the consequent drop of the forefoot favors a PF of the first metatarsal as the IAMTJ dorsiflexes during the final phase of gait, generating greater pressure on the first and fifth MTH. This movement will generate a more effective windlass mechanism in feet with lateralized loads.

Bojsen-Moller [25] divided the foot into two axes across which it can propel a transverse axis (through the first and second MTH) and an oblique axis (third, fourth and fifth MTH). Propulsion through the transverse axis was produced by medializing the physiological loads to effect the propulsive moment; the medial side of the foot was locked, acting as a rigid lever ready to propel effectively. When this physiological medialization was not generated, the medial area of the foot acted as a flexible and unstable lever, which would generate an apropulsive gait that must be carried out via the oblique axis, which was accompanied by the blocking and levering of the lateral column. When there was a limitation of IMTJ, the forces could be lateralized to be able to perform the propulsion from an oblique axis.

Roukis et al. [26] concluded that an alteration in the normal functioning of the first radius, such as hypermobility or insufficiency, could generate a lateralization of GRF and increased pressure on the lesser metatarsals in adult subjects; Menz et al. [4] indicated that if asymptomatic IMTJ was present, there was an increased pressure peak under the hallux, but if this area caused pain, an antalgic gait was adopted which shifted the pressure peak to the side. According to our findings, the functional limitation we studied in children does not result in pain, so the antalgic gait that can occur in adults with HL is unlikely to be reproduced in school-aged patients with HL.

Bryant et al. [16] divided the foot into ten regions to classify and differentiate the pressure peaks in each of them in healthy feet, with LH and HR. In our research, we divided the foot into seven regions as this seemed more appropriate for children with mostly small feet and it would be difficult to differentiate between the third and fourth MTHs and lesser toes.

Furthermore, Visscher et al. [27] reported that clinical identification methods commonly used during biomechanical studies were often subjective and time-consuming and used a pressure platform. Therefore, research in this field should continue to validate biomechanical studies and ensure that results can be unified and homogeneous for researchers.

Agostini et al. [28] performed a study with a sample size of 85 children, 42 male and 43 female, aged between 6 and 7 years old. According to the measurements obtained with a pressure platform and regarding the height of the children, they concluded that their findings must be considered as a key factor to obtain gait data because the sensors did not obtain all the information and did not establish a correct relationship such as speed. Related to our findings, there were data that we were unable to obtain due to the platform used, and that would have been very useful for comparing the propulsion phase between both groups, with respect to having been able to check the speed–time factor.

Beurskens et al. [29] demonstrated that in children who consciously or unconsciously perform dual tasks during dynamics, gait velocity, stride length and cadence may be reduced. Thus, variability may increase during platform pressure measurements in children. For this study, there were no dual tasks while collecting pressure platform measurements, but it could be that they were distracted by uncontrolled or unnoticed momentum. For this reason, further studies should be conducted with this population to validate the shape, spacing, length and other characteristics that may vary during pressure measurement with a platform.

Several limitations were found in our research. One of the main limitations was age. Our research was conducted in a school-age population aged between 7 and 12 years old and pubertal stage changes were not considered between sexes, especially in females. In future research, the influence of pubertal stages should be taken into account. On the other hand, in our research all the subjects had good health condition with a normal BMI, but nevertheless physical activity and body conditions such as the lean mass of the body and lower limb were not considered; for future research, these factors could be interesting for the final outcomes. Regarding our research, we assert that this method of assessment obtained positive results as an instrument for the quantitative assessment of HL in school-aged children. These findings can be explained by biomechanics; given that there is functional limitation of movement in the IMTJ in a school-age population, there is no pain and therefore nothing to compensate for and thus no antalgic gait. In addition, the limitation is not sufficiently rigid to transfer loads to other more mobile joints by lateralizing the loads; therefore, pressure peaks are produced in the hallux and IMTH during the moment of propulsion, generating inefficient propulsion with this toe acting as a rigid lever. Therefore, it is necessary to carry out a good individualized podiatric study for each patient to diagnose the pathology and apply the appropriate treatment in each case. Moreover, in children, limitation of movement in any joint implies blockages of a higher or lower level, increasing the likelihood of a future pathology.

## 5. Conclusions

Dynamic baropodometry is a useful tool in biomechanical studies because it facilitates the identification of gait abnormalities. School-age subjects with hallux limitus during gait exhibit more average peak plantar pressure in the heel and less peak average plantar pressure in the third and fourth metatarsal heads as compared to healthy children aged between 7 and 12 years old.

## Figures and Tables

**Table 1:** Sample sociodemographic characteristics across groups.

Sample Characteristics	Total Group (*n* = 100) Mean ± SD (Range)	HL (*n* = 50) Mean ± SD (Range)	Healthy (*n* = 50) Mean ± SD (Range)	*p*-Value
Age (years)	9.62 ± 1.37 (7–12)	9.68 ± 1.29 (7–12)	9.56 ± 1.46 (7–12)	0.607 †
Weight (kg)	37.84 ± 11.04 (20.50–90.00)	37.12 ± 8.53 (23.30–56.60)	38.57 ± 13.13 (20.50–90.00)	0.809 †
Height (cm)	140.37 ± 10.75 (118.0–176.00)	140.10 ± 9.54 (121.00–167.00)	140.64 ± 11.92 (118.00–176.00)	0.885 †
BMI (kg/m^2^)	18.83 ± 3.68 (11.00–40.00)	18.64 ± 2.68 (14.00–25.00)	19.02 ± 4.48 (11.00–40.00)	0.803 †
Sex (male/female)	50/50 (50.0/50.0)	22/28 (44.0/56.0)	28/22 (56.0/44.0)	0.317 ‡
Foot size	35.99 ± 2.67 (28.00–43.0)	36.09 ± 2.20 (31.0–43.0)	35.88 ± 3.08 (28.00–42.0)	0.665 †

Abbreviations: kg, Kilogram; cm, Centimeter; m^2^, Square Meter; % Percentage; SD, Standard Deviation; N, Number. † Mann–Whitney U test was used. ‡ Fisher exact test was used. In all the analyses, *p* < 0.05 (with a 95% confidence interval) was considered statistically significant.

**Table 2:** Principal outcomes measured for the control group and subjects with HL.

Variables	Total Group (*n* = 100) Mean ± SD (Range)	HL (*n* = 50) Mean ± SD (Range)	Healthy (*n* = 50) Mean ± SD (Range)	*p*-Value
Left surface area (cm^2^)	88.59 ± 19.63 (37–141)	89.54 ± 18.12 (51–127)	87.64 ± 21.17 (37–141)	0.634 †
Right surface area (cm^2^)	89.72 ± 19.99 (29–135)	90.38 ± 19.58 (44–125)	89.06.11 ± 20.57 (29–135)	0.796 †
Left maximum peak pressure (kPa)	1792.72 ± 479.28 (17.0–3566.0)	1789.92 ± 308.41 (1160–2503)	1795.51 ± 307.43 (17–3566)	0.751 †
Right maximum peak pressure (kPa)	1757.88 ± 482.93 (12–3854)	1759.10 ± 366.86 (262–2616)	1756.66 ± 580.19 (12–64)	0.697 †
Left average peak pressure (kPa)	1089.47 ± 862.36 (46–9310)	1162.89 ± 1188.17 (716–9310)	1016.06 ± 282.42 (46–1945)	0.560 †
Right average peak pressure (kPa)	1000.88 ± 237.94 (24–1906)	1002.08 ± 180.93 (683–1516)	999.69 ± 285.74 (24–1906)	0.890 †
Body weight in hallux (left/right) (%)	11/14 (11.0/14.0)	10/8 (20.0/16.0)	1/6 (2.0/12.0)	0.127 ‡
Body weight in first metatarsal head (left/right) (%)	9/7 (9.0/7.0)	8/5 (16.0/10.0)	1/2 (2.0/4.00)	0.354 ‡
Body weight in second metatarsal head (left/right) (%)	11/23 (11.0/23.0)	4/12 (8.0/24.0)	7/11 (14.0/22.0)	0.156 ‡
Body weight in third & fourth metatarsal heads (left/right) (%)	37/22 (37.0/22.0)	11/7 (22.0/14.0)	26/15 (52.0/30.0)	0.031 ‡
Body weight in fifth metatarsal head (left/right) (%)	1/1 (1.0/1.0)	0/0 (0.0/0.00)	1/2 (2.0/4.00)	1.325 ‡
Body weight in midfoot (left/right) (%)	0/1 (0.0/1.0)	0/0 (0.00/0.00)	0/1 (0.00/2.00)	1.432 ‡
Body weight in heel (left/right) (%)	31/31 (31.0/31.0)	17/18 (34.0/36.0)	14/13 (28.0/26.0)	0.023 ‡

Abbreviations: kPa, kilopascals; cm^2^, Square Centimeter; %, Percentage; SD, Standard Deviation; N, Number. † Mann–Whitney U test was used. ‡ Fisher exact test was used. In all the analyses, *p* < 0.05 (with a 95% confidence interval) was considered statistically significant.

## Data Availability

The dataset supporting the conclusions of this article is available upon request at claudia.cuevas@udc.es, Research, Health, and Podiatry Group (Department of Health Sciences, Faculty of Nursing and Podiatry, Industrial Campus of Ferrol, Universidade da Coruña).
